# Massachusetts Dental Society

**Published:** 1885-01

**Authors:** W. E. Page


### MASSACHUSETTS DENTAL SOCIETY.

Reported for the Independent Practitioner by W. E. Page, D. M. D.

The twentieth annual meeting of this society was held in the rooms of the Boston Medical Library, Dec. 11th and 12th, 1884.

The meeting was called to order by the president, Dr. D. M. Clapp, and the minutes of the last meeting were read and approved.

The annual address was delivered by Dr. E. G. Leach, of Boston. Papers were also read by Dr. G. F. Eames, upon "The Intimate Nature of Inflammation;" by Dr. J. H. Kidder, upon "Experiments;" by Dr. E. B. Hitchcock, upon "The Effects of Sugars and their Compounds in the Oral Cavity;" and by Dr. H. C. Merriam, upon "Gutta Percha and Its Uses in Operative Dentistry."

E. H. Bradford, M. D., exhibited a patient with congenital anchylosis of the jaw.

Dr. R. R. Andrews discussed different process of tooth formation, and exhibited twenty illustrative micro-photographs.

Dr. H. O. Marcy, chairman of the Committee on Legislation of the Massachusetts Medical Society, explained the object of the appointment of his committee, and presented a copy of the act to be laid before the legislature, and asked co-operation. It was voted that a special committee of five be appointed by this society, with full power to take such action as they deemed best regarding dental legislation. The committee appointed consisted of Drs. D. B. Ingalls, J. G. W. Werner, G. F. Eames, E. H. Smith and J. H. Kidder.

Dr. W. E. Page, Committee on Diploma, presented a steel plate, a fac-simile of the old diploma, which was accepted.

It was voted that the semi-annual meeting be held in Worcester, in June, in conjunction with the Connecticut Valley Dental Society.

The officers elected for the ensuing year are:

*President*—Dr. J. F. Adams, Boston.

*First Vice-President*—Dr. J. G. Stevens, Lynn.

*Second Vice-President*—Dr. E. B. Hitchcock, Boston.

*Secretary*—Dr. W. E. Page, Boston.

*Treasurer*—Dr. Edward Page, Charlestown.

*Librarian*—Dr. R. R. Andrews, Cambridge.

*Executive Committee*—Drs. G. F. Eames, J. K. Knight, E. C. Leach, J. G. W. Werner, F. A. Cooke.

